# Understanding and meeting the needs of those using growth hormone injection devices

**DOI:** 10.1186/1472-6823-6-5

**Published:** 2006-10-11

**Authors:** Hervé Dumas, Paris Panayiotopoulos, Dorothy Parker, Vincent Pongpairochana

**Affiliations:** 1 Serono International SA, 15 bis Chemin des Mines, Geneva, CH-1202, Switzerland; 2 Fast Forward Research Ltd, Crown House, Manchester Road, Wilmslow, Cheshire SK9 1BH, UK; 3 Laboratoires Serono SA, Patients Care Technologies, Coinsins, CH-1267, Switzerland

## Abstract

**Background:**

Recombinant human growth hormone (r-hGH) is used to treat: growth hormone deficiency in children and adults; children born small for gestational age; Turner's syndrome; and chronic renal failure. r-hGH is administered by daily subcutaneous injection and may be given using a number of different administration devices. The aim of this survey was, firstly, to identify which attributes of an r-hGH administration device are considered most important to physicians, teenage patients, parents of young children requiring GH and nurses who have experience of r-hGH administration, and, secondly, to determine how they rate existing devices in each of these key attributes.

**Methods:**

The opinions of 67 individuals with experience in r-hGH administration were captured in discussion sessions. Parents, physicians and nurses were asked to rate 19 device attributes by completing a questionnaire, and to rank four different r-hGH administration devices (including a conceptual electronic device) in order of preference.

**Results:**

Reliability, ease of use, lack of pain during injection, safety in use, storage, and number of steps in preparation before use, during use and after were considered to be the five most desirable attributes of an r-hGH administration device. An electronic device was preferred to an automatic, multi-dose injection device, a needle-free injection device or a manual, ready-to-use, disposable injection device.

**Conclusion:**

In the opinion of physicians, nurses and parents using r-hGH injection devices, an ideal device must combine reliability with simplicity, while delivering treatment with minimal pain. An electronic device, which combines many of the most useful features of existing devices with novel functions, was the preferred option for r-hGH administration.

## Background

Approximately 1 in 4000 children are born every year with growth hormone deficiency (GHD) [1]. GHD causes short stature, low growth velocity, excess subcutaneous fat and delayed skeletal maturation [1], which have a considerable impact on physical and psychological functioning [2]. Adults with untreated GHD also have an increased cardiovascular risk [3]. Replacement therapy using exogenous GH has been used successfully since the 1950s to treat children (and more recently adults) with GHD [4,5].

Early preparations of GH were extracted from human pituitary glands, but its use was discontinued in 1985 following the diagnosis of four cases of Creutzfeldt-Jakob disease in patients who had received GH [6]. Later in the same year, the first recombinant form of human GH (r-hGH) became available. Initially, r-hGH was produced using genetically engineered bacterial cells (*Escherichia coli*) [7], but in 1987 a mammalian cell-derived r-hGH preparation (produced by murine C127 cells) was introduced [8]. With the advent of a new, unlimited source of GH, researchers were able to explore the use of GH for other conditions associated with growth retardation or metabolic dysfunction. Today, GH is used to treat not only GHD in both children and adults [4,5] but also a number of other disorders, including Turner's syndrome [9], chronic renal failure [10], and children born small for gestational age [11].

To achieve optimal therapeutic results with GH, continuous, long-term adherence is essential. However, all existing r-hGH products are administered subcutaneously, usually on a daily basis, and this can lead to problems with adherence [12]. Therefore, it is important that devices used for r-hGH administration are convenient and acceptable to patients. In particular, a large proportion of the patients who require r-hGH therapy are children, so an r-hGH administration device must be child-friendly. The optimal device must be simple enough for a child to operate easily and safely. The option of a hidden needle or a needle-free device may also be particularly useful in making the administration of r-hGH more acceptable to children. The first commercially available preparations of r-hGH were injected using a standard syringe, but novel administration devices – pre-filled syringes, manual injector pens, auto-injectors, injectors with hidden needles and needle-free devices – have since been introduced in an attempt to increase dosing accuracy and adjustability, ease of use, convenience, adherence, simplicity, and patient-friendliness [13-21].

Despite these advances in r-hGH administration device design, there is still scope for improvement. None of the current devices are pain-free, and developments that reduce pain or the patient's perception of pain (psychological pain) are also likely to increase adherence and acceptance of therapy. In addition, it may be possible to improve existing features or to introduce new, useful features, such as pre-set dosing programmed by the physician, administration/cartridge replacement reminder alarms or adjustable injection speeds. Some of these functions may help to improve adherence but would need to be incorporated in a way that did not compromise the simplicity of the device.

The aim of this survey was, firstly, to identify which attributes of an r-hGH injection device are considered most important by physicians, teenage patients, parents and nurses who have experience of GH administration, and, secondly, to determine how these participants rate existing devices in each of these key attributes, and to use this to identify any unmet needs in GH injection devices.

## Methods

Individuals were recruited for this survey in France, Germany, Italy, the UK and the USA. Participants included prescribing physicians (endocrinologists and paediatric endocrinologists, no more than two from the same hospital, with recent experience of treating patients aged under 18 years with r-hGH), nurses involved in patient training and support (community- or hospital-based, no more than two from the same hospital), teenage patients (aged between 13 and 15 years) self-injecting r-hGH using a delivery device, and parents injecting their children (aged under 14 years) with r-hGH. Recruitment of patients/parents was facilitated with the assistance of medical professionals (in Europe) or patient associations (in the USA).

Group discussions involving three to six individuals were conducted to assess opinions relating to r-hGH administration device attributes. These discussions lasted 2–3 hours, were audio and video recorded, and were viewed by up to five experts in a professional viewing room. Where participants were unable to attend group discussions (e.g. issues with geographical logistics), individual face-to-face interviews were carried out (audio-recorded). During these discussions and interviews, participants were made aware of the current device options, asked to brainstorm their desired alternative devices and to suggest improvements.

Physicians, nurses and parents were also asked to complete a questionnaire assessing which attributes they felt to be the most important in a device for r-hGH administration. The participants first spontaneously identified the key device attributes that were important for them. These attributes were then compared with a pre-existing list and the participants were free to add extra attributes from this list, if they wished. The participants were then asked to score each of the selected attributes. A total of 19 attributes were assessed to evaluate the importance of ergonomics, functionality and the psychological impact of such a device: number of steps in preparation, no need for reconstitution, ease of releasing trapped air, automatic injecting, drug pre-loaded, disposable device, multi-dose, ease of use, reliability, pain during injection, level of noise during injection, design aspects, position of release button, size, weight, level of physical strength required to operate, ease of grip, ease of storage, safety in use and in storage. Each attribute was rated from 0 (not important at all) to 10 (extremely important). The questionnaire also asked participants to rank the performance of existing r-hGH injection devices that they had used previously for each of these attributes, from 0 (not well at all) to 10 (extremely well). It was not considered appropriate for the younger patients to complete a questionnaire, so only the questionnaire results of parents, nurses and physicians are presented below under Results. The children's views were expressed spontaneously and key aspects were probed for (e.g. their views on ease of use, size, etc.). These children were also asked to produce drawings of desired features/new devices during the group sessions.

Three r-hGH administration devices were demonstrated during the discussion sessions: an automatic, multi-dose injection device (one.click™, Serono, Geneva, Switzerland), a needle-free injection device (cool.click™, Serono, Geneva, Switzerland) and a manual, ready-to-use, disposable injection device (FlexPen^®^, Novo Nordisk, Dublin, Ireland). The concept of an electronic device was also introduced. Devices were ranked in order from 1 (preferred choice) to 4 (least preferred choice). In addition, individual and consensus opinions relating to the attributes of each device were captured during these discussions.

Mean scores were calculated for each attribute assessed in the questionnaire. Mean scores were also calculated for each attribute, grouped by participant type or by country.

## Results

This survey assessed the views of 67 individuals: 19 endocrinologists (Germany, France, Italy and the USA), 18 nurses (UK, Germany, France and the USA), 12 patients (UK and Germany) and 18 parents (Germany, France and the USA). A total of 13 group discussions were conducted, as well as seven face-to-face interviews (in France and the USA).

**Figure 1:**
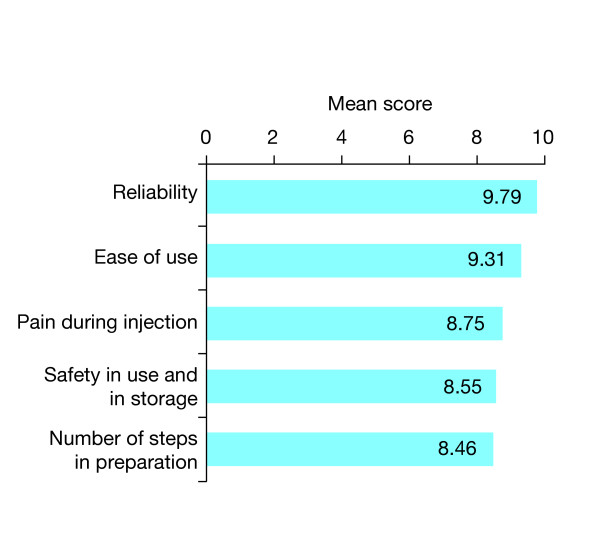
**Most important device attributes**. Mean scores for the five attributes of a recombinant human growth hormone administration device considered most important by participants in the survey. Device attributes (19 in total) were assessed in the questionnaire completed by physicians, nurses and parents.

Of the 19 key device attributes that were rated, participants considered the top five most important features of an injection device to be:reliability, ease of use, lack of pain during injection, safety in use and in storage, and the number of steps in preparation before use, during use and after (Figure 1). Parents, nurses and physicians all regarded reliability as the most important device attribute and ease of use as the second most important device attribute. Participants in most countries (Germany, France and the USA) considered reliability to be the most desirable device attribute, while lack of pain during injection and the number of steps in preparation were ranked the most important device attributes by participants in the UK and Italy, respectively.

**Figure 2:**
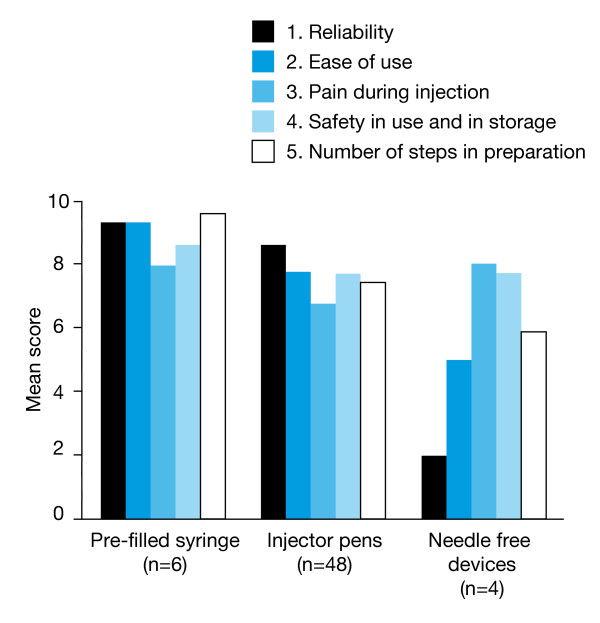
**Comparative ratings for devices used previously**. Comparative ratings for recombinant human growth hormone injection devices used previously by participants in the survey. Device attributes were assessed in the questionnaire completed by physicians, nurses and parents. Ratings are shown as mean scores for the five device attributes considered most desirable by survey participants.

Nearly all participants (58/67, 87%) had previous experience with the Genotropin Pen^® ^(Pharmacia, Stockholm, Sweden) and nearly half (32/67, 48%) had previous experience with the HumatroPen^® ^(Eli Lilly, Indianapolis, USA) and/or the NordiPen^® ^(30/67, 45%). Eight of the r-hGH injection devices used previously by participants were rated using the questionnaire. Mean scores for these devices (grouped according to device type) in the five attributes considered most desirable are shown in Figure 2.

**Figure 3:**
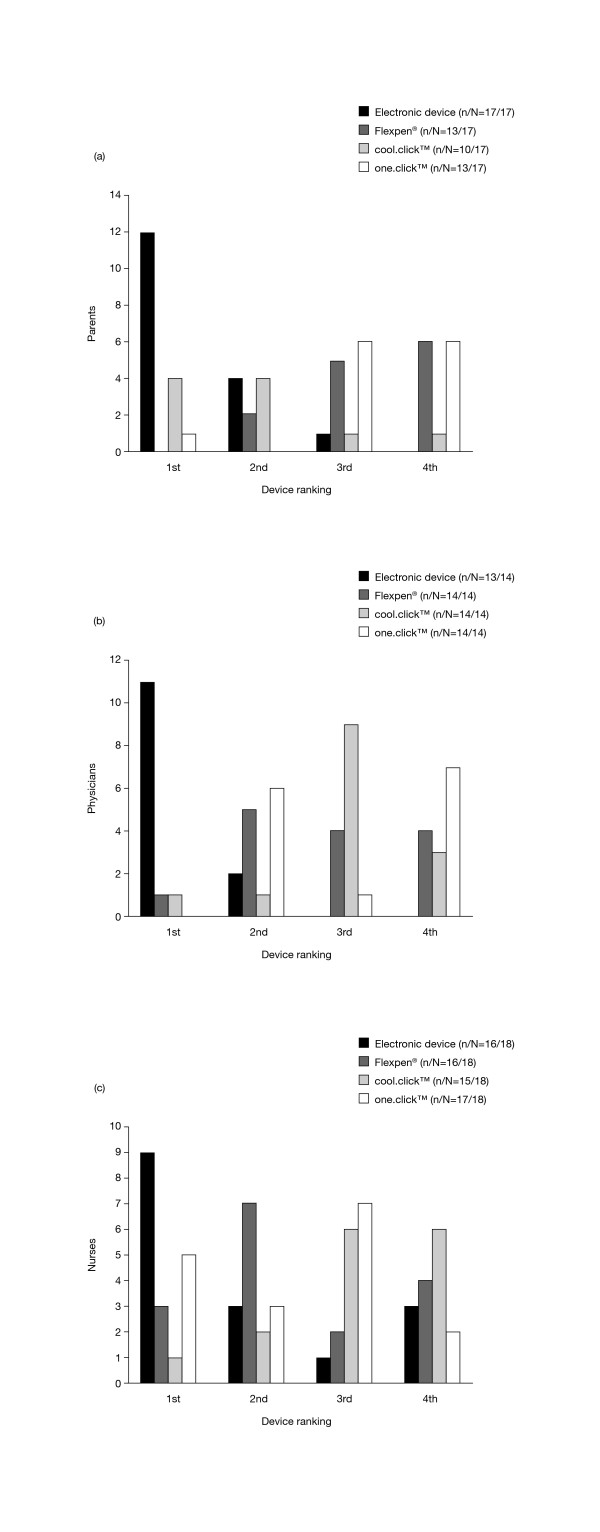
**Preferences for devices demonstrated or introduced**. Preferences expressed by parents (a), physicians (b) and nurses (c) for each of the four recombinant human growth hormone administration devices demonstrated (Flexpen^®^, cool.click™, one.click™) or introduced (electronic device) during discussion sessions. Devices were ranked in order from 1 (preferred choice) to 4 (least preferred choice). n/N refers to the number of individuals ranking each device out of the total number surveyed. **(a) **– Preferences expressed by parents. **(b) **– Preferences expressed by physicians. **(c) **– Preferences expressed by nurses.

Of the four device options that were demonstrated/introduced during discussions, the electronic device was considered to be the preferred device option by parents (12/17, 71%), physicians (11/13, 85%) and nurses (9/16, 56%; Figure 3). Of the remaining three devices, parents generally preferred the cool.click™ needle-free device, while physicians and nurses preferred the Flexpen^® ^and/or the one.click™ device.

## Discussion

The nurses, physicians and parents questioned in this survey considered reliability to be the most important attribute that they required from an r-hGH injection device. Device reliability encompasses not only the ability of the device to deliver the injection of r-hGH each time it is used, but also to confirm that the correct dose has been administered. To ensure reliability, an r-hGH administration device must be robust enough to withstand daily use and handling by young children. It is understandable that reliability is considered to be so important to those using r-hGH injection devices because, if a device stops functioning, the patient/parent may need help to get it repaired, and this must be done quickly to ensure continuity of treatment. Repairs may involve driving to the pharmacy/hospital, which is both inconvenient and time-consuming. For some devices, technical support is available over the phone to help patients/parents rectify problems with their device at home.

Of the devices they had used previously, the participants generally considered pre-filled syringes and auto-injector pens to be highly reliable (mean score: 8.6–9.3). Only four participants had previous experience with needle-free devices and considered the reliability of these devices to be relatively low (mean score: 2.0).

Participants considered 'ease of use' to be the second most important attribute for an r-hGH injection device; ease of use is particularly important when the device is being used by a child or adolescent. A device with only a few steps in preparation and for which dose adjustment/resetting is straightforward will be simple to use. For children, the size and weight of the device, as well as the strength required to administer the injection will also be important. Of the injection devices they had used previously, participants found pre-filled syringes and auto-injector pens very easy to use. The four patients who had previous experience with needle-free devices considered them to be moderately easy to use.

Lack of pain was considered by participants to be the third most important attribute for an r-hGH injection device. The pain and perceived pain associated with the administration of a treatment may directly influence a patient's adherence and acceptance of the therapy. Therefore, it is important to minimise the pain and perceived pain associated with the r-hGH injection process. A patient's experience of pain (both real and perceived) is related to both injection technique and needle quality [22]. Of the devices they had used previously, the participants associated all three types of r-hGH administration device with similar levels of pain. These pain levels were rated as moderate (mean score: 6–7) and, therefore, this highlights a key area in which improvements could have a significant positive impact on the patient. It may be possible to introduce device design features that can reduce both the real pain (e.g. finer needles and adjustable injection speeds) and the perceived pain (e.g. needle-free or hidden-needle options and noiseless operation) experienced by the patient.

When asked to consider which type of device would best meet their needs, parents, nurses and physicians in our survey preferred the electronic device more than an automatic, multi-dose injection device, a needle-free injection device or a manual injection device. An electronic device is also likely to be popular with children and teenagers who are familiar with modern hand-held devices such as mobile phones and computer games.

An electronic device has the potential to meet many of the key patient needs highlighted by this survey. For example, adherence aids (e.g. reminder messages and tracking functions) could be incorporated, and these are particularly desirable as they may help to increase the clinical effectiveness of GH therapy. Multiple sensor and precision cartridge detection will provide reliable confirmation to the patient or physician that the correct dose has been delivered. Pre-programmed dosing and automatic needle attachment/detachment will both make the device easier to use. Auto-injection using a permanent hidden needle will help to reduce the patient's perception of pain associated with the injection. In addition, such a device has the potential to offer novel features such as adjustable injection speeds, noiseless operation, and a cartridge replacement alarm.

## Conclusion

Our survey indicates that, in the opinion of physicians, nurses and parents using r-hGH injection devices, an ideal future device must combine reliability with simplicity, while delivering treatment with minimal pain. An electronic device was considered the preferred option for r-hGH administration, compared with existing automatic injection devices or prefilled syringes. An electronic device combines many of the most useful features of existing r-hGH administration devices with novel additional functions, enabling it to meet the key needs of those who use these devices.

## Competing interests

HD, PP and VP are employees of Serono International SA. DP is an employee of Fast Forward Research Ltd who conducted the survey for Serono International SA, Geneva, Switzerland.

## Authors' contributions

PP, DP and VP were involved in survey conception and design. Data were acquired by DP, who interpreted it with the help of PP and VP. HD helped to draft the manuscript, which was critically reviewed by HD, PP and VP. All authors have read and given final approval for the manuscript to be published.

## Pre-publication history

The pre-publication history for this paper can be accessed here:


